# Reply: Coin-shaped epithelial lesions following an acute attack of erythema multiforme minor with confocal microscopy findings

**DOI:** 10.4103/0301-4738.77016

**Published:** 2011

**Authors:** Kalpana Babu, Vinay R Murthy, Veeresh P Akki, Venkatesh C Prabhakaran, K R Murthy

**Affiliations:** Vittala International Institute of Ophthalmology and Prabha Eye Clinic and Research Center, Bangalore, Karnataka, India

Dear Editor,

We thank Jain *et al*.[1] for their interest in our article.[2] We do agree that these lesions do improve with topical steroids. What is important here is the presence of additional systemic features like skin eruptions or involvement of a mucosa. In our patient, there were skin eruptions and painful buccal mucosal ulcers following a viral illness. Herpes simplex virus-induced erythema multiforme (EM) minor constitutes 15-60% of EM minor. The treatment is a combination of acyclovir and oral steroids.[3]

Treatment with oral steroids along with oral acyclovir is definitely justified in our case. In contrast, we do see cases of EM minor with only ocular involvement in the form of coin-shaped lesions which will definitely improve only with topical steroids just like the case described by Jain *et al*.[1] As we have described the confocal microscopy findings speculating an immunological mechanism in the development of these coin-shaped lesions on the cornea, these lesions will definitely respond to topical steroids.
